# Juvenile idiopathic arthritis in two tertiary centres in the Western Cape, South Africa

**DOI:** 10.1186/1546-0096-10-35

**Published:** 2012-10-10

**Authors:** Kate Weakley, Monika Esser, Christiaan Scott

**Affiliations:** 1 Dept of Paediatric Rheumatology, School of Child and Adolescent Health, University of Cape Town, Red Cross Childrens Hospital, Klipfontein Rd, Rondebosch, Cape Town, South Africa; 2 Department of Paediatric Rheumatology and Immunology, Tygerberg Hospital, Stellenbosch University, Tygerberg, South Africa

**Keywords:** Juvenile idiopathic arthritis, South Africa, Clinical characteristics, Functional disability

## Abstract

**Background:**

Juvenile idiopathic arthritis (JIA) is a disease that shows wide variations between differing populations. Since the recent international consensus on classification criteria, JIA has been widely described in many countries and population groups. There has been almost no data that describes JIA in an African, specifically Sub-Saharan African, setting. Therefore, the aim of this study is to describe disease characteristics, disease course, and functional disability in two tertiary centres in the Western Cape, South Africa and compare the findings to other JIA populations.

**Methods:**

Eighty-six children were recruited during random clinic visits to rheumatology clinics at Tygerberg and Groote Schuur Hospital between April 2010 and April 2011. Children were diagnosed using International League of Associations for Rheumatology (ILAR) 2001 classification criteria. Consent was obtained and medical records examined. The Childhood Health Assessment Questionnaires (CHAQ) and visual analogue scales (VAS) for pain and general well-being were completed and all children were examined by a researcher in conjunction with a paediatric rheumatologist. HIV status as well as tuberculosis disease and treatment were investigated.

**Results:**

A total of 86 children were enrolled. Eight children were excluded (2 HIV arthropathy, 1 TB arthritis, 1 SLE, 4 with insufficient data), leaving a total of 78 patients. There was an equal female to male ratio-39 males and 39 females. There were 6 systemic JIA patients (7.69%), 17 persistent oligoarthritis (21.79%), 4 extended oligoarthritis (5.12%), 11 polyarthritis rheumatoid factor (RF) positive (14.10%), 21 polyarthritis RF negative (26.9%), 1 psoriatic arthritis (1.28%), and 18 enthesitis-related arthritis (23%). The median CHAQ for the group was 0.5 (IQR 0.1-1.25), the median VAS for pain was 18 mm (IQR 4–42) and median VAS for general well-being was 25 mm (IQR 3–49). Enthesitis-related arthritis and polyarthritis disease subtypes in this South African population may be more common than seen in JIA populations described in northern Europe, India, United Kingdom, and Turkey.

**Conclusion:**

This Western Cape South African JIA population appears to have a different profile of JIA than what has been described elsewhere. Enthesitis-related arthritis and polyarthritis disease subtypes appear to be more prevalent. There are also significant challenges in this setting such as later presentation to pediatric rheumatologists, different disease characteristics, and variable disease courses.

## Background

Juvenile idiopathic arthritis (JIA) is a poorly described disease in South African children. According to the International League of Associations for Rheumatology (ILAR) [1], JIA is defined as arthritis that begins before the 16th birthday and persists for at least 6 weeks, other conditions being excluded. The ILAR classification has, in recent years, been used all over the world to delineate JIA disease characteristics in various populations and nationalities. Included in these recent studies are large multicentre trials and smaller studies from both developed and developing countries [2-6].

South Africa is a country that has limited resources in paediatric rheumatology. South Africa has very few trained pediatric subspecialists caring for children with rheumatic disease and educating medical students and pediatric trainees. In South Africa, there is also a high burden of infectious diseases (HIV, TB) as well as social diseases (poverty, malnutrition). These diseases demand a great amount of attention and resources, and therefore the amount of resources, education, and research into rheumatic diseases such as JIA has been very limited.

JIA is the most common rheumatic disease in children [7]. JIA is an important cause of short and long term disability in children with decreased daily function and quality of life [8,9].

Studies in developed countries have estimated a prevalence of JIA that varies between 0.07-4 per 1000 children [10,11], yet there is very little consensus on the exact prevalence of JIA. As evidence increasingly shows, it is likely that JIA prevalence is underestimated. In the South African setting, there is relatively poor access to healthcare, complex economic migration, and large socioeconomic discrepancies. Therefore no accurate prevalence figures are available for South Africa.

JIA is a disease that shows variation in expression between different ethnicities [12]. As these variations may provide insight into the cause of JIA, there is substantial interest in these differences in JIA in various parts of the world and various population groups [2-6]. The only South African study that described disease characteristics of JIA was a study done in the Kwazulu Natal region of South Africa in 1984 [13] using the 1977 European League Against Rheumatism criteria for juvenile chronic arthritis [14]. This classification divided JIA into polyarticular, pauciarticlar and systemic subtypes. A predominance of polyarticular JIA and equal male to female ratio among patients with juvenile chronic arthritis was described. This study was the only study to describe JIA in South Africa in the last 30 years. Unfortunately the study’s usefulness now is limited by the use of the old European classification.

Cape Town is an area with a unique population profile in terms of ethnicity and race. The population of children (0–17 years old) in Cape Town is 1.7 million [15]. Of these children approximately 34.9% are Black African, 44% are Coloured, 1.8% are Asian and 19.3% are White [16]. In the South African ethnic context, “Coloured” refers to a population of mixed ancestry with some Black, Asian and European heritage. Cape Town is a diverse city with 2 tertiary hospital centres. The first is Groote Schuur Hospital associated with the University of Cape Town and the second is Tygerberg Hospital, which is associated with Stellenbosch University. Both of these hospitals service the Western Cape of South Africa as tertiary referral centres and both have paediatric rheumatology clinics which collaborate with each other. Thus the aim of this study is to describe the disease characteristics and functional disability in a sample of children with JIA from 2 tertiary centres in Cape Town, South Africa and compare these patterns to those described by studies in the UK, India, Europe, US, Japan, and Turkey.

## Methods

### Setting

Patients were recruited from the Groote Schuur and Tygerberg Hospital Paediatric Rheumatology clinics between March 2010 to April 2011. These clinics service approximately 400 children with arthritis. There are 5 clinics per week.

### Participants

Participants were patients attending the above clinics who were diagnosed with juvenile idiopathic arthritis as per the ILAR 2001 definition [1]. The ILAR classification is routinely used in the clinic. Patient records were checked to ensure that patients fit all the criteria to be classified. In patients who had attended clinic for a longer period of time, records were reviewed and discussed with a paediatric rheumatologist to classify the child. New patients were classified according to his/her presenting features and history of disease gained from the parents or the child. Patients were recruited at random visits when a researcher was available. No limitation was set on whether it was the patient’s initial or a return visit.

### Procedures

#### Data sheet

A researcher collected patient data on a data collection sheet including demographics, disease subtype, and routine blood results. Blood tests were drawn only if necessary for routine medical care and previous relevant blood results were collected from the case notes. It was not routine to perform blood tests for ANA, RF or HLAB27 at every visit due to resource limitations. ANA was only done routinely in children who presented with oligoarthritis, HLAB27 was only done in those with other features of ERA, and RF was only done in children with polyarthritis. It must be noted that the majority of ANA blood tests were ELISA tests as HEp 2 immunofluoescent ANA studies were only available at Tygerberg Hospital.

### Childhood health assessment questionnaire

A Childhood Health Assessment Questionnaire (CHAQ) was filled out by the parent, or when capable, by the child. The parent’s version of the CHAQ incorporates a doubly-anchored horizontal 100 mm visual analogue scale (VAS) for the assessment of the child’s overall well-being (with anchors of ‘0 = very well’ and ‘100 = very poor’) and a doubly-anchored horizontal 100 mm VAS for the assessment of the intensity of the child's pain (with anchors of ‘0 = no pain’ and ‘100 = very severe pain’) [2]. The CHAQ is a well validated international tool and has been used in many recent JIA trials [17]. The CHAQ was translated into Zulu, Xhosa and Afrikaans for the purposes of this study but not yet formally validated.

### Joint count

All participants were then examined by a researcher and a paediatric rheumatologist together. A joint count was performed and recorded on 2 joint diagrams, one for limited joints (limited range of movement) and one for active joints (swollen or limited and tender) [1].

### Analysis

The data was analysed using STATA software and a number of statistical tests were applied.

### Ethics

Ethics approval was granted by both the University of Cape Town and Stellenbosch University ethics committees. All procedures of the study were explained in detail to the parents and patients. All parents and, where possible, capable children (age over 16) signed consent forms prior to enrolling in the study. It was clearly explained that should the patients not wish to participate in the study, it would not affect their ongoing care. No additional blood tests/procedures or radiological tests were done for the purposes of the study. Confidentiality was ensured for all participants. No conflict of interest was declared. Any stationary/transport costs were minimal and covered personally by the researcher. Patients were not paid for their participation or offered any incentive.

## Results

A total of 88 children were enrolled in the study. Ten children were excluded leaving a total of 78 patients. Two patients refused consent. Four patients were excluded due to insufficient clinical data collection. Of these 4 excluded patients, 1 patient with oligoarthritis had an insufficient clinical examination recorded; 1 patient with oligoarthritis had an incomplete consent form and 2 patients (1 polyarthritis, 1 oligoarthritis) had blood results that could not be found. Two children were diagnosed with HIV-related arthritis and excluded. One child initially had arthritis and then developed definite SLE and was excluded. One child was later diagnosed with arthritis due to tuberculosis and had to be excluded.

There was an exactly equal female to male ratio: 39 males and 39 females. The median age at diagnosis was 8 years of age (IQR 4–10). The median current age was 13 years old (IQR 8–17). There were 6 systemic JIA patients (7.69%), 17 persistent oligoarthritis patients (21.79%), 4 extended oligoarthritis (5.12%), 11 polyarthritis rheumatoid factor (RF) positive (14.1%), 21 polyarthritis RF negative (26.9%), 1 psoriatic arthritis (1.28%), and 18 enthesitis-related arthritis (ERA) (23%). There were no patients who were classified as undifferentiated (Table 1).

**Table 1:** Variables per type

**Type**	**No**	**Percent**	**Age at onset**	**Current age**	**CHAQ**	**Pain VAS (mm)**	**General VAS (mm)**	**Active joints**	**Limited joints**	**ESR**
**Systemic**	6	7.69%	2	5.5	0.437	10.5	12	3	0	58.5
**Persistent oligo**	17	21.9%	3	8	0.375	19	23	1	1	9
**Oligo Extended**	4	5.12%	4	8	0.563	21	34.5	5.5	4.5	5
**Poly RF+**	11	14.1%	10	15	1	20	30	3	9	42
**Poly RF-**	21	26.9%	8	13	0.875	21	27	2	6	12.5
**ERA**	18	23%	11.5	17	0.125	14	14	1	1	12
**Total Cohort**	78		8 (4–10)	13 (8–17)	0.5 (0–1.125)	18 (4–42)	25 (3–49)	2 (1–4)	2 (1–6)	14 (5–42)

All values are expressed as medians as they show non normal distribution overall. The values in the brackets are interquartile ranges, which are given for the overall sample.

A median CHAQ, pain VAS, General VAS, active joint count, limited joint count, ESR and CRP, was calculated for each subtype as can be seen in Table 1. All data is displayed as medians. As there was only 1 psoriatic arthritis patient, it has been excluded from this Table.

Sixty-seven patients had ANA tests done. Only 3 patients were ANA positive (1 oligoarthritis, 2 oligoextended). Of these 3 patients, 2 were positive by the immunofluorescent HEp 2 ANA assay and 1 by use of the ELISA technique. The majority of the ANA tests performed were done using the ELISA tests, which is not as reliable. Eighteen patients were HLA-B27 positive, all of whom were ERA patients by the ILAR classification (i.e., they also had enthesitis or the other criteria for ERA). Eleven patients were rheumatoid factor positive, all of whom had polyarthritis.

## Discussion

The population of children with JIA in our study cohort has a different profile from studies done in other parts of the world. Table 2 summarizes some of the differences between various populations worldwide in some recent studies that use the ILAR classification from developed and developing countries. Even though our data is not normally distributed, the mean values are included in brackets for comparison purposes to the other study cohorts. All of these studies were done in tertiary centres with paediatric rheumatologists.

**Table 2:** Comparison of data

**Study**	**Morocco**^**4**^	**Turkey**^**6**^	**Printo**^**2**^**W. Europe**	**Printo**^**2**^**E. Europe**	**Printo**^**2**^**Latin America**	**India**^**5**^	**South Africa**
**N**	80	196	2102	668	397	235	78
**Age current**	10.85*	8.8*	9.4*	11.4*	10.7*		13(12.4*)
**Age at onset**	7.53*	7.0*	5.4*	6.8*	6.6*	12	8(7.3*)
**Female**	59%	47%	83%	72%	68%	41.7%	50%
**Systemic**	26%	15.3%	16.4%	23%	28.5%	8%	7.69%
**Polyarthritis**	31.5%	37.2%	32%	33%	40.6%	29%	40.9%
**Poly RF+**		6.6%				12%	14%
**Poly RF-**		30.6%				17%	26.9%
**Oligo Persistent**	37,5%	24.4%	29.5%	31.6%	21.9%	17%	21.8%
**Oligo extended**	5%	9.6%	21.5%	12%	9%	4%	5%
**ERA**		10.3%				36%	23%
**Psoriatic**		1%				1%	1.28%
**Undifferentiated**		2.5%				5%	
**VAS pain(mm)**	28.4*		28*	23*	27*		18(24.9*)
**VAS general(mm)**			25*	28*	24*		25(28.4*)
**Limited joints**	1.78*		5.9*	8.6*	10.1*		2(6.51*)
**Active joints**	4.39*		5.3*	6.7*	7.1*		2(3.85*)
**CHAQ**	0.84*		0.7*	0.6*	0.8*		0.5(0.73*)
**ESR**	28*		30.7*	27.7*	31.2*		14 (25.1*)

Median values represented unless * marks mean value.

This data from tertiary centres may skew the results in 2 ways. Patients who attend a paediatric rheumatology clinic may have more severe disease than those in the community who never make it to the tertiary centre. These milder forms of JIA have a higher chance of going into remission and may not need to be referred to tertiary centres. This may cause a skewed perception that JIA is more severe in any area of South Africa. In addition, more severe subtypes may be over-represented in the sample. The various studies also had differing methodologies which must be noted as a limitation to the comparison below. Table 3 summarizes the different methods used. Limitations of our study include sample bias, for the reasons mentioned above. Another limitation is sample size, which is small compared to other groups.

**Table 3:** Comparison of methods

**Study**	**Setting**	**Method**	**No. patients**	**Time period of data collection**
**Morocco**^**4**^	Tertiary	Cross sectional prospective	80	18 months
**Turkey**^**6**^	Tertiary	Retrospective record review	196	
**PRINTO**^**2**^	Tertiary	Multicentre case control cross sectional	3167	
**India**^**5**^	Tertiary	Longitudional cohort	235	11 years
**South Africa**	Tertiary	Prospective cross sectional	78	1 year

### Age

Out of the above studies, the present data of South African centres has the oldest mean current age (12.4 years) and the 2nd youngest mean age of onset (7.3 years). One of the reasons for these results could be that the present studies’ patients are being referred to treatment centres relatively long after their initial onset of symptoms. In our socioeconomic scenario, we would suggest that one of the most likely reasons for this is a delay in diagnosis, leading to a delay in attempting to induce disease remission, and therefore an older group of children who actively attend our clinics. Another reason for the older mean current patient age may be that we have a relatively poor transition to adult care compared to other places. It would appear that there is a longer disease course before presentation to a South African paediatric rheumatology centre.

### Gender

Next to India (58.3%) and Turkey (53%), the present data has the 3rd highest rate of male JIA (50%), with all of the other regions having a female predominance as is classically described in western populations [5,6]. There is a difference in gender distribution per subtype. If ERA is excluded them from the our group, there is a 65% female predominance.

### Polyarthritis

In the studies compared in Table 2, our data shows the highest rate of rheumatoid factor positive polyarthritis (14%), and the second highest rate of rheumatoid factor negative polyarthritis (26.9%) next to Turkey (30.6%) [6]. These are significant trends and are in keeping with previous data that describes African population groups as having a higher risk of the polyarthritis subtype of JIA [12,13,18]. A limitation that must be mentioned in the present study is that the ILAR criteria require at least 2 positive RF assays to be done at least 3 months apart in the first 6 months of the disease in order to diagnose RF positive polyarthritis [1]. Due to constraints on resources, this obtaining of 2 RF titers is not standard practice in our clinics, and one positive or negative assay was considered sufficient to classify a patient with polyarthritis. This has been a difficulty with the ILAR classification, especially in poorly resourced settings. This difficulty is mentioned in the Indian study [5] and in a study of Nordic children [19]. Thus we believe that the RF positive polyarthritis patients may be overrepresented in our polyarticular subtype as a child with one positive assay was classified as RF positive polyarthritis without having a repeat test.

### Oligoarthritis

Lower rates of oligoarthritis (21.8% oligo-persistent, 5% oligo-extended) are seen in the present cohort than described in Western populations. This is in keeping with previously described data that shows that non-European populations have a decreased relative risk of suffering from oligoarthritis [12]. This may be a true reflection of a decreased prevalence or it may reflect that these patients have less obviously severe disease and would be underrepresented in a tertiary centre due to the constraints on awareness and resources mentioned above. These oligoarthritis children with less arthritis may do comparatively well in the community and not be felt to need tertiary referral. There is also a very low rate of ANA positivity. A limitation that must be mentioned is that there are technical issues with the available laboratories, with the majority of ANA testing being done using ELISA studies and only a small proportion of our patients using the HEp2 immunofluorescent ANA studies test. It has been shown that ELISA ANA testing often have fewer ANA positives than HEp2 immunofluorescense testing [20,21].

### Enthesitis related arthritis

The present data describes the second highest rate of ERA (23%), second only to the Indian cohort (36%) [5]. It is much higher than the ERA rates reported in a recent UK longitudional cohort study (7%) and the Turkish study above (10.3%) [3,6]. The large PRINTO series states that there were too few a number of ERA patients, so they were excluded from further consideration [2].This higher rate of ERA is an interesting finding as ERA has not been previously associated with an African population. It has been described as being more prevalent in Asian populations and Indian populations [5,12]. This may be due to the unique population mix that is prevalent in the Western Cape region of South Africa. The Western Cape has a different population to the rest of South Africa, with a higher prevalence of coloured people (44%) compared to black (34.9%) [16]. This is not representative of the rest of the country where the majority of the population is black. The coloured population has mixed ancestry with some Asian and European heritage which may account for the high levels of ERA. It may also be due to a founder effect from the initial small immigrant population groups in the Cape.

### Psoriatic

There was only 1 patient with psoriatic arthritis, which may be due to psoriatic arthritis subtype being a rarer subtype in our setting. It was difficult obtaining a family history of psoriasis in our patients and this historical challenge may have decreased psoriatic patients. The prevalence of psoriasis in general in South Africa is not known.

### Undifferentiated

There were no patients classified as undifferentiated arthritis. This may be a reflection of small sample size, or the above mentioned referral bias.

### Functional disability and outcomes

In Table 2, it is shown that our cohort has a comparable mean ESR, CHAQ, pain and general wellbeing to the other groups.

## Conclusions

JIA in this study population has a different profile compared to other international JIA studies. However, our study is limited by sample size and sample bias. There is an equal male to female predominance. Both RF positive and negative polyarthritis are more common than has been described elsewhere. There is a higher rate of ERA than has been previously described in Africa. Lower rates of oligoarthritis are described. Disease diagnosis is often later than elsewhere due to socioeconomic factors, confounding diseases such as TB, lack of JIA awareness, and inadequate resources in paediatric rheumatology. There are difficulties with the ILAR classification in our setting, specifically regarding the requirement of 2 rheumatoid factor tests.

## Abbreviations

JIA: Juvenile idiopathic arthritis; IQR: Interquartile range; ILAR: International league of associations for rheumatology; HIV: Human immunodeficiency virus; TB: Tuberculosis; PRINTO: Paediatric international trials organisation; UK: United kingdom; CRP: C reactive protein; ESR: Erythrocyte sedimentation rate; HB: Haemoglobin; Plt: Patelets; ANA: Anti-nuclear antibody; RF: Rheumatoid factor; HLAB27: Human leukocyte antigen B27; AST: Aspartime transaminase; ALT: Alanine transaminase; ELISA: Enzyme linked immunosorbent assay; CHAQ: Childhood health assessment questionairre; CI: Confidence interval; VAS: Visual analogue scale; ERA: Enthesitis related arthritis; CAPS: Childhood arthritis prospective study.

## Competing interests

We declare that there are no financial competing interests. This study was undertaken as a thesis component of a MMed degree for Kate Weakley.

## Authors’ contributions

KW-Primary Investigator and author. ME-Contributing author. CS-Supervisor and author. All authors read and approved the final manuscript.
